# The challenge of recruiting patients into a placebo-controlled surgical trial

**DOI:** 10.1186/1745-6215-15-167

**Published:** 2014-05-13

**Authors:** Kristoffer B Hare, L Stefan Lohmander, Ewa M Roos

**Affiliations:** 1 Institute of Sports Science and Clinical Biomechanics, University of Southern Denmark, Campusvej 55, 5230 Odense M, Denmark; 2 Department of Orthopedics, Slagelse Hospital, Ingemannsvej 18, 4200 Slagelse, Region Zealand, Denmark; 3 Department of Orthopedics, Clinical Sciences Lund, University of Lund, Paradisgatan 2, 221 00 Lund, Sweden

**Keywords:** Feasibility, Placebo, Surgery, Recruitment, Trial

## Abstract

**Background:**

Randomized placebo-controlled trials represent the gold standard in evaluating healthcare interventions but are rarely performed within orthopedics. Ethical concerns or well-known challenges in recruiting patients for surgical trials in general have been expressed and adding a placebo component only adds to this complexity. The purpose of this study was to report the challenges of recruiting patients into an orthopedic placebo-controlled surgical trial, to determine the number of patients needed to be screened and allocated in order to include one participant into the trial, and to identify reasons associated with participation in a placebo-controlled randomized surgical trial.

**Methods:**

Data were extracted from an ongoing placebo-controlled randomized controlled trial (RCT) on meniscectomy versus placebo surgery. We calculated the number of patients needed to be screened in order to include the required number of participants into the RCT. Participating patients were asked about their rationale for joining the study and which type of information was most useful for deciding upon participation.

**Results:**

A total of 476 patients entered the screening group, of which 190 patients fulfilled the inclusion and exclusion criteria. 102 patients declined to participate in the study due to various reasons and 46 were later excluded (no meniscus lesion on the magnetic resonance imaging scan or withdrawn consent). A total of 40 patients were finally included in the RCT. To include one patient into the RCT, 11.9 individuals needed to be screened. A total of 69% of participating patients considered the oral information to be the most important and the most common reason for participating was the contribution to research (90%).

**Conclusions:**

Patients are willing to participate in an orthopedic placebo-controlled surgical trial. Oral information given by the surgeon to the patient and the contribution to research are important aspects to enhance patient recruitment.

**Trial registration:**

ClinicalTrials.gov NCT01264991, registered 21 December 2010.

## Background

Randomized controlled trials (RCTs) represent the gold standard in evaluating healthcare interventions. The randomization of treatment and blinding of group allocation to the investigator and participants, possibly by use of a placebo, reduces bias [1]. Though this design is considered gold standard in therapeutic trials this has not been the case within the field of surgery. Ethical concerns [2,3] or well-known challenges in recruiting patients for surgical trial in general [4,5] have been expressed and adding a placebo component only adds to this complexity.

We are not aware of any studies on the challenge of recruiting patients for a placebo-controlled orthopedic trial, specific screening procedures for identifying eligible patients, or motivation of patients to participate in a placebo-controlled orthopedic trial. One study on feasibility and acceptance showed that an orthopedic placebo-controlled trial could be conducted in principle, albeit with difficulty [6]. The challenge of recruitment for an orthopedic RCT comparing rehabilitation plus early surgery with rehabilitation plus optional later surgery for an acute ACL (anterior cruciate ligament) tear have been reported, [7] but similar reports from placebo-controlled trials are lacking. Earlier placebo-controlled orthopedic studies have reported a recruitment rate between 35 and 71% [8-10] but no information exists on patient’s preferences and determinants of willingness to participate in an orthopedic placebo-controlled study.

Arthroscopic partial meniscectomy (APM) is the most commonly performed orthopedic procedure, carried out on 1 million patients annually in the United States [11]. The mean age in most studies is around 50 years of age [8,12-14], indicating the vast majority of procedures being performed in patients with a degenerative disease. Both meniscus injury and meniscectomy are associated with the development of knee osteoarthritis (OA) [15-17]. Previous studies in patients with or without concomitant OA have found APM to be no better than, or have no additional benefit in comparison to, placebo surgery, lavage, optimized non-surgical treatment, or exercise [8,12-14].

This report describes the challenges of recruiting patients into an ongoing multicenter RCT [18] comparing APM to a placebo surgery of degenerative meniscus tears in a younger age group (between 35 and 55 years of age) at an earlier stage of disease. We provide the number of patients needed to be screened (NNS), and the number of patients needed to be allocated (NNA), in order to include the required number of participants into the RCT. We further identify the reasons associated with participation in a placebo-controlled randomized surgical trial by asking why patients were willing to participate and which type of information was most useful for deciding upon participation.

## Methods

We recruited and screened patients aged between 35 and 55 years, having a magnetic resonance imaging (MRI) confirmed medial meniscus lesion and at least two months of knee pain without any previous significant trauma. Eligible patients were randomized to placebo surgery or APM after having agreed to participate in the RCT and after providing signed informed consent (Table 1).

**Table 1:** Inclusion and exclusion criteria in the RCT of APM versus placebo surgery of degenerative meniscus tears

**Inclusion criteria**	
1.	Knee pain > 2 months without significant trauma
2.	MRI confirmed medial meniscus lesion
3.	Age 35-55
4.	Eligible for outpatient surgery
**Exclusion criteria**	
1.	Need for acute surgery, i.e. locking knees, high energy trauma
2.	Symptoms from other musculoskeletal disorder overriding symptoms of the knee
3.	Grade 3 or 4 knee OA on the Kellgren-
4.	Lawrence classification
5.	Knee surgery within the last 2 years
6.	BMI > 35
7.	Ischemic heart disease
8.	Diabetic late complications
9.	Thrombophilia
10.	Pregnancy
11.	Unable to speak Danish
12	Drug or alcohol abuse

APM, arthroscopic partial meniscectomy; BMI, Body Mass Index; OA, osteoarthritis; RCT, randomized controlled trial.

Both APM and the placebo surgery were performed under general anesthesia but only skin incisions equivalent to two standard portals were performed in the placebo group.

The complete design and methodology of the study have been published [18]. The study is approved by the Research Ethics Committee of Region Zealand, Denmark, and is consistent with the Declaration of Helsinki.

### Screening strategies

Patients referred from general practitioners were screened for eligibility by the principal investigator, an orthopedic surgeon in residency. If eligible, oral and written information were given about the study including a 12-minute DVD to view at home. A few days later the patients were contacted by telephone and provided temporary consent by phone, and if willing to participate they were referred for an MRI to confirm a meniscus lesion. If their MRI confirmed a medial meniscus lesion the patient provided written consent, was included in the study, and signed up for surgery if still willing to participate. The strategy of asking patients to participate before performing an MRI was chosen since an MRI is not routinely performed before an arthroscopy in Denmark.

### Patient information

Patients eligible for an MRI all received the same oral and written information. The oral information was given by the principal investigator in a standardized way. Patients were informed of the nature of a degenerative meniscus lesion, the treatment options, and hereunder surgery. They were informed about the lack of evidence for the effects of meniscus surgery in older age groups. They were then informed of the lack of trials in their age group, the need for a study, the general concept of the placebo effect, and the design of the current study, including information that placebo surgery would mean that no intervention on their meniscus tear would be performed.

The written information was identical to the oral information apart from formal information about study origin, study investigators, information on possible adverse events (most common infection and deep venous thrombosis) and other treatment modalities (such as exercise).

A 12-minute DVD was given to all eligible patients prior to the MRI to further ensure uniform dissemination of information to all patients. The video described the background for the study, the amount of involvement for participating and showed interviews of three different orthopedic surgeons with extensive experience within knee surgery giving their view on the condition and arthroscopic meniscus surgery.

### Statistics

The number needed to screen (NNS) and the number needed to allocate (NNA) are concepts used and described in previous studies [7,19,20]. The NNS was calculated by dividing the number of patients screened for eligibility with the number of patients included in the trial. Similarly the NNA was calculated by dividing the number of allocated patients with the number of included patients. All patients eligible for inclusion were regarded as allocated. The NNS and NNA provided an estimate of how many patients were needed to be screened and allocated to include one patient into the trial. Multiplied with the *a priori* determined sample size, the NNS gives an estimate of how many patients needed to be screened and the NNA an approximation of the total number of eligible patients necessary.

All participating patients were asked about their rationale for joining the study: wanting to contribute to research, wanting an operation no matter which type, or other, with room for elaboration. They were also asked which kind of information had been most beneficial in deciding whether or not to participate: the oral information given by the orthopedic surgeon, the written information, or the 12-minute DVD. The proportions of these answers were calculated and reported with 95% confidence intervals (95% CI).

## Results

**Figure 1:**
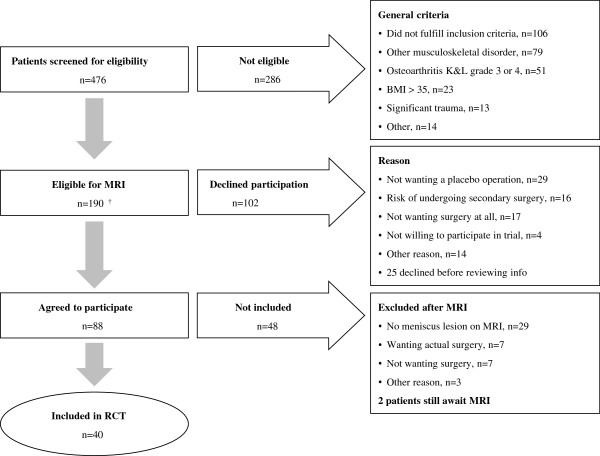
**Flowchart of the recruitment process in this RCT.** †4 patients underwent an MRI which was negative before being informed of the study. BMI, Body Mass Index; RCT, randomized controlled trial.

Since the start of the study, 476 patients with a suspicion of medial meniscus injury referred from their general practitioner entered the screening group. A total of 190 patients fulfilled the inclusion and exclusion criteria and were thus eligible for an MRI. However, of these, 102 patients declined to participate in the study. More specifically, 77 did not wish to participate after reviewing the patient information, the reasons being: a) not wanting placebo surgery (38%), b) the risk of undergoing a secondary operation if allocated to the placebo group (21%), and c) not wanting surgery at all (19%). A small number (5%) did not want to participate in any scientific study and 17% had other reasons, mostly work-related. A total of 25 patients declined participation before reviewing the patient information and their reasons for declining participation were not collected. In addition, 46 were excluded after no visible meniscus tear was seen on MRI. Finally, 40 patients were included in the RCT (Figure 1). To include one patient into the RCT, 11.9 individuals with a suspected meniscus lesion needed to be screened. Similarly, the NNA was 4.8 individuals eligible for inclusion (prior to MRI) needed in order to include one patient in the RCT.

Of the 40 included patients, the most common reason for participating was the contribution to research (90% (80 to 100, 95% CI)) compared to other reasons (10% (0 to 20, 95% CI), *P* < 0.001). A total of 69% (54 to 84, 95% CI) of participating patients considered the oral information from the orthopedic surgeon as the most important compared to the written information and DVD (31% (16 to 46, 95% CI), *P* < 0.05).

## Discussion

There is a great need for randomized controlled, including placebo-controlled, orthopedic trials. The most common reasons for this lack of placebo-controlled surgical RCTs include ethical issues and difficulty in recruiting patients. The recruitment of patients into orthopedic RCTs is a well-recognized challenge [4,5] and adding a placebo component to the trial potentially further complicates recruitment.

In our placebo-controlled study we showed that 46% of patients fulfilling clinical eligibility criteria were willing to participate in a placebo-controlled arthroscopy trial, fully aware they would have general anesthesia and possibly surgery without any real intervention being performed. These patients weighted the oral information given as more important than the written information and the 12-minute DVD when deciding whether to participate or not. They also reported that the contribution to science was the main reason for participating, which is similar to other studies [21-23].

A NNS of 11.9 is high, compared to other trials of orthopedic surgery. Buchbinder *et al.*[9] reported a NNS of 6 for their placebo-controlled RCT of vertebroplasty and Frobell *et al.*[7] reported an NNS of only 5.5 in their RCT study comparing rehabilitation plus early surgery with rehabilitation plus optional later surgery for an acute ACL injury. One other study by Katz *et al.*[12] comparing APM in combination with physiotherapy or physiotherapy alone reported a NNS of 41.1, indicating only a minority of patients meeting the inclusion criteria. The NNS is markedly affected by the screening strategy, pathoanatomy and clinical inclusion criteria, and planned interventions, and hence will vary between different trials for different conditions. In this study the high NNS was influenced by the fact that the screening was made on a broad population of patients referred from general practitioners less experienced in knee examination and before any imaging was performed. Thus patients with symptoms and clinical signs not related to an MRI-verified meniscus injury constituted a large part of the screening population. Performing an MRI after clinical screening meant that more patients had to be screened. Future clinical trials of meniscus surgery may consider performing an MRI earlier during the screening process to lower the number of patients seen by the clinician. Degenerative meniscus tears are difficult to detect and assess by clinical examination alone [24] and in the present study an MRI failed to show a meniscus tear in 33% of eligible symptomatic patients willing to participate, confirming a poor correlation between clinical signs and MRI findings in this patient group [16,25].

The NNA of 4.8 was also higher than other comparable studies: the NNA for the vertebroplasty trial [9] was 2.1, for the ACL treatment trial [26] 1.6, and for another placebo-controlled study of medial degenerative tears by Sihvonen *et al.*[10] the NNA was only 1.4. Despite this, it still took five years in five different centers to include 146 patients. That is approximately six patients included per year per center. This could suggest that definition of eligibility and/or timing of consent were different than from our study. The NNA in the study by Katz *et al.*[12] was 3.8, mainly because approximately 60% of the patients declined to participate. In our study also more than half the patients declined to participate. This is no different from other placebo-controlled trials on already established surgical procedures and emphasizes the importance of evaluating the effect from surgical interventions in a similar fashion as for other therapeutic trials [27], alas prior to introducing a new surgical procedure. Another reason for the high NNA was that 17 patients withdrew their initial consent after their MRI. Half of these patients withdrew consent because, although having a positive MRI finding, their symptoms had regressed and they no longer experienced a need for surgery, and the other half because they would not risk receiving a placebo operation.

## Conclusions

In conclusion we have shown that patients are willing to participate in an orthopedic placebo-controlled surgical trial. Challenges remain to improve screening procedures for an improved feasibility, and pilot studies are critical for a realistic assessment of NNS and NNA. We recommend that when recruiting patients for a placebo-controlled surgical trial, focus should be on the oral information given by an orthopedic surgeon and the patient’s contribution to science should be emphasized.

## Abbreviations

ACL: Anterior cruciate ligament; APM: Arthroscopic partial meniscectomy; CI: Confidence interval; DVD: Digital video disc; MRI: Magnetic resonance imaging; NNA: Number needed to allocate; NNS: Number needed to screen; OA: Osteoarthritis; RCT: Randomized controlled trial.

## Competing interests

The authors declare that they have no competing interests.

## Authors’ contributions

KBH participated in the design of the study, was responsible for data collection and drafted the manuscript. LSL participated in the design of the study and helped to draft the manuscript. EMR participated in the design of the study and helped to draft the manuscript. All authors read and approved the final manuscript.
